# Genetic evidence of regulatory gene variants of the *STAT6, IL10R* and *FOXP3* locus as a susceptibility factor in uncomplicated malaria and parasitaemia in Congolese children

**DOI:** 10.1186/1475-2875-12-9

**Published:** 2013-01-08

**Authors:** Felix Koukouikila-Koussounda, Francine Ntoumi, Mathieu Ndounga, Hoang V Tong, Ange-Antoine Abena, Thirumalaisamy P Velavan

**Affiliations:** 1 Institute of Tropical Medicine, University of Tübingen, Wilhelmstrasse 27, Tübingen, Germany; 2 Fondation Congolaise pour la Recherche Médicale, Brazzaville, Republic of Congo; 3 Faculty of Health Sciences, Marien Ngouabi University, Brazzaville, Republic of Congo; 4 Centre d’Etudes sur les Ressources Végétales, Brazzaville, Republic of Congo; 5 Unité de Recherche sur le Paludisme, Hôpital de Base de Makélékélé, Brazzaville, Republic of Congo

**Keywords:** *Plasmodium falciparum*, Tregs, *FOXP3*, *IL10RA*, *STAT6*

## Abstract

**Background:**

Regulatory T cells (Tregs) are a subset of T cells that play an important role in modulating T effector responses during infectious challenges. The aim of this study was to evaluate possible associations between regulatory gene polymorphisms and the risk of uncomplicated malaria and the control of *Plasmodium falciparum* parasite density levels.

**Methods:**

Twelve regulatory single nucleotide polymorphisms (SNPs) in the promoter regions of *FOXP3* (ss270137548, rs11091253), *IL10RA* (rs56356146, rs7925112), *IL10RB* (rs8178433, rs8178435, rs999788), *STAT6* (rs3024941, rs3024943, rs3024944) and *TNFRSF18* (ss2080581728, rs3753344) were genotyped in a cohort of Congolese children. Studied subjects were followed up (passively) during one year. The children who experienced one or several clinical episodes were genotyped as “uncomplicated malaria” group (n=179) and those children who did not experience any episode were genotyped as “asymptomatic children” group (n=138).

**Results:**

The prevalence of *rs3024944CC* genotype of *STAT6* was significantly higher in the group of asymptomatic children compared to that of uncomplicated malaria (*P*=0.003). Similarly, the minor allele *rs3024944C* was more prevalent in the group of asymptomatic children (*P*=0.019). Two novel SNPs were observed including *-163T/G* (ss491228441) in *IL10RA* gene and *-163C/T* (ss491228440) in *TNFRSF18* gene. The genotype *ss491228441TT* and the minor allele *ss491228441G* of the *IL10RA* were more frequent in the group of asymptomatic children (*P*=0.006 and *P*=0.007, respectively). The genotype *rs11091253CT* of the *FOXP3* was associated with high parasite density levels. In addition, a new promoter *IL10RA* variant (ss491228441) contributes to shield against mild malaria.

**Conclusion:**

The study indicated that the *STAT6* promoter polymorphism rs3024944 was associated with uncomplicated malaria, whereas the *FOXP3* promoter variant rs11091253 was associated with significant *P. falciparum* parasitaemia levels. These genetic data may contribute to the understanding of molecular mechanisms that regulate immune response to *P. falciparum* infections.

## Background

Malaria is one of the major causes of morbidity and mortality in tropical and sub-tropical areas. Approximately 216 million clinical cases of malaria occurred worldwide in 2010, resulting in almost 655,000 deaths [1]. Sub-Saharan Africa remains the region with the highest burden of the disease, accounting for nearly 91% of global malaria deaths. Malaria is caused by protozoan parasites of the genus *Plasmodium* and *Plasmodium falciparum* being the most virulent species. *Plasmodium falciparum*-infected individuals show different clinical phenotypes, which range from asymptomatic infections to severe forms of malaria [1]. Several investigations demonstrated that immune gene polymorphisms in human host, such as those having an impact on expression of T regulatory cells (Treg), contribute to the variability of malaria phenotypes [2,3]. The generation of Tregs has been demonstrated to modulate the immune response in malaria infection as well as in other infectious diseases [4-8]. A few of the Tregs are induced in response to infectious challenge and the others are considered as natural regulators [4]. Expression of Tregs is well known to be regulated by number of human host genes, such as the forkhead box P3 (*FOXP3*), interleukin 10 receptors alpha and beta (*IL10RA* and *IL10RB*), the signal transducer and activator of transcription 6 (*STAT6*), and the tumour necrosis factor receptor superfamily member 18 (*TNFRSF18*) [9].

*FOXP3* belongs to the family of transcription factors that play a role in various cellular processes and is described as an important regulator of natural Tregs development and function [10-12]. In humans, mutations in the *FOXP3* gene are known to be associated with immune dysregulation polyendocrinophathy and enterophathy X-linked syndrome (IPEX) caused by lack of Tregs [13]. *IL10RA* and *IL10RB* are two receptor chains forming the human interleukin 10-receptor (*IL10R*) which belongs to the class II cytokine receptors [14]. The major role of *IL10RA* precursor is to mediate high affinity and ligand binding and subsequent signal transduction, whereas the *IL10RB* precursor is believed to contribute only in signalling process [15]. Reports have demonstrated that mutations in the genes encoding *IL10RA* and *IL10RB* precursors of the IL10R annul the IL10 mediated immunomodulatory signalling and are strongly associated with inflammatory bowel disorders [16]. Recently combined promoter haplotypes of the *IL10RA* and *IL10RB* genes have been shown to be associated with protection against severe malaria in Gabonese children [14]. The *STAT6* gene, which belongs to the STAT family of transcription factors, has been reported to mediate various cytokine-induced responses. Recent reports have shown that *STAT6* gene polymorphisms play crucial roles in controlling helminth infection. More precisely, a promoter variant rs324013 was found to be associated with the control of *Schistosoma haematobium* in a Dogon population (where *S. haematobium* is endemic) in Mali [17]. Similarly, the variant rs324015 was associated to the control of infection levels in Chinese children from an endemic area for *Ascaris lumbricoides*[18]. *TNFRSF18*, also known as glucocorticoid-induced TNF receptor, is a member of TNF superfamily, which plays a key role in the co-stimulatory process for T cell activation for both conventional and CD25^+^CD4^+^ T cells. In addition, *TNFRSF18* expressed on Tregs was demonstrated to play a crucial role in the maintenance of peripheral tolerance [9]. Therefore, all these findings suggest that mutations in *FOXP3*, *IL10RA*, *IL10RB*, *STAT6* and *TNFRSF18* may lead to various immunopathological reactions in auto-immune disorders and/or parasitic infections.

In a previous investigation, regulatory SNPs in the promoter region of *FOXP3*, *IL10RA*, *IL10RB*, *STAT6* and *TNFRSF18* genes were identified in the Gabonese population in Central Africa. These identified SNPs were further validated for their allelic gene expression using transient transfection assays [14,19-21]. Against this background, in the present study, the possible relationship between the reported SNP variants and risk of having uncomplicated *P. falciparum* malaria was investigated. For this purpose, this study utilized a case-control cohort of Congolese children, who were followed up (passively) during one year for surveillance of clinical malaria cases, comprising a group of children who experienced one or several uncomplicated malaria episodes and that of children who had no clinical episode during the follow up. As a second objective, the impact of these SNPs carriage on the *P.falciparum* parasite density levels was assessed.

## Methods

### Study area, study population and ethical consideration

The study was carried out in three districts (Kinsana, Mbouono and Ngoko) of Makélékélé health division in the southern area of Brazzaville. These districts are semi-urban with about 6,000 inhabitants and located along the Congo River. Malaria is transmitted throughout the year with *P. falciparum* being the predominant species and *Anopheles gambiae* the main mosquito vector [9]. This study, as part of a cohort study for malaria surveillance in the study area (Ndounga *et al,* unpublished), is a community-based case-control study. The study population consisted of children aged one to nine years permanently living in the study area. All the study individuals belong to Bantu ethnicity and had equivalent risk for infection. Children were recruited between April and June 2010 and passively followed up during one year. At the time of enrolment 4 ml of whole blood were collected from all children and a thick and thin blood smears were performed. The inclusion criteria were: absence of clinical malaria in the previous two weeks and one week after enrolment (an axillary temperature of <37.5°C, no disease symptoms such as muscular pain and headaches), negative thick blood smear or positive blood smear with less than 5,000 parasites per microlitre of blood (p/μl), and not being homozygous for sickle cell trait. During the follow up, at any time, when a child was suspected to have fever or any malaria-related symptoms, parents or guardians were invited to present the child at the health centre for clinical examination. In case of malaria (positive thick and thin blood smears and axillary temperature ≥37.5°C), blood sample was collected, parasite density determined and children were treated with artesunate-amodiaquine or artemether-lumefantrine. No data were available about the present or past history of helminth infection.

The ethical approval was given by the Institutional Ethics Committee for Research on Health Sciences of the Republic of Congo. Written informed consent was obtained from parents or guardians of children.

### Microscopic examination

Thick and thin blood films were stained with 10% Giemsa for 15 min and read by two independent, competent microscopists to determine the parasite density and *Plasmodium* species. Asexual parasites were counted against 200 leucocytes and expressed as the number of asexual parasites per μl, assuming the leucocytes count of 8,000/μl of blood.

### DNA extraction

Genomic DNA was extracted from 200 μl of peripheral whole blood samples obtained from patients and asymptomatic children using the commercially available QIAamp DNA Blood Mini Kit (Qiagen, Hilden, Germany) following the manufacturer’s instructions and stored at -20°C until use.

### SNP genotyping

The promoter region of each of the five targeted genes in all the samples was genotyped for the promoter SNP variants that revealed altered gene expression as reported in earlier investigations. Those SNPs are: *-794C/G* (ss270137548) and *-738C/T* (rs11091253), for the *FOXP3* gene; *-185C/T* (rs56356146), and *-116T/C* (rs7925112) for the *IL10RA* gene; *-978T/G* (rs8178433), *-754A/G* (rs8178435), and *-740C/T* (rs999788) for the *IL10RB* gene; *-3728C/G* (rs3024941), *-3430G/A* (rs3024943), and *-3387G/C* (rs3024944) for the *STAT6* gene; and *-265C/T* (ss2080581728), and *-199C/T* (rs3753344) for the *TNFRSF18* gene. Polymerase chain reaction (PCR) amplifications were carried out in 20 μl reaction volumes with 5 ng of genomic DNA, 1X PCR buffer (20 mM of Tris-HCL pH 8.4, 50 mM of KCL, 1.5 mM of MgCl_2_), 0.2 mM of dNTPs, 0.5 mM of each primer and 1 U of Taq DNA polymerase (Qiagen, Hilden, Germany). Thermal cycling parameters are mentioned in Table 1. All the PCR products were purified with the PCR DNA Purification Kit (GE Healthcare Europe GmbH) and 1 μl of the purified product was directly used as template for sequencing using the BigDye terminatorv.2.0 cycle sequencing Kit (Applied Biosystems, USA) on an ABI 3130 DNA sequencer, according to the manufacturer’s instructions. Sequence-specific primers designed and used for amplifications and sequencing are listed in Table 1. Polymorphisms were identified by assembling the sequences with respective reference sequences obtained from SNPper database using Codon code Aligner 4.0 software and were reconfirmed visually from their respective electropherograms.

**Table 1:** Primers used for genotyping and sequencing

**Gene ID**	**Chr.**	**Primer sequences (Forward/reverse)**	**Size**	**Thermal Conditions**
*FOXP3*	X	5^′^-AGGCTGAGGGCCTCAGAAGCATC-3^′^*	429 bp	94°C(5mn), [94°C(15S), 61°C(1mn) 72°C(1mn)]x35, 72°C(10mn)
		5^′^-CTCCAGGCCTCAGTTTCCCTATAG-3^′^		
*IL10RA*	11	5^′^-GGGAAGGAAAGGGAGGGGTGGC-3^′^*	241 bp	94°C(2mn), [94°C(30S) 61°C(30S),72°C(30S)]x35, 72°C(2mn)
		5^′^-GCGCGCCTCCAGCTACCCTTG-3^′^		
*IL10RB*	21	5^′^-AAGGATGAGGCTGTGAGGAGG-3^′^*	401 bp	94°C(2mn), [94°C(30S) 61°C(30S),72°C(1mn)]x35, 72°C(2mn)
		5^′^-CAAGCAGAGGGAAGTGAATGCG-3^′^		
*STAT6*	12	5^′^-GGCGTGTCTCAGTGTTTACCCC-3^′^*	530 bp	94°C(2mn), [94°C(30S) 61°C(30S),72°C(1mn)]x30, 72°C(2mn)
		5^′^-GTCCCCCTCAAAAGCATCAGC-3^′^		
*TNFRSF18*	1	5^′^-TCCATGGTTGAGGCTCTCTGG-3^′^*	293 bp	94°C(2mn), [94°C(30S) 61°C(30S),72°C(1mn)]x35, 72°C(2mn)
		5^′^-GTGTGAGGAGGGGGTGTAGAC-3^′^		

Chr: Chromosome, *: Sequencing primers, bp: Base pairs, mn: minutes, S: seconds.

### Statistical analysis

In this study, children without any reported malaria episode during the follow-up period were considered as asymptomatic and were classified in a group as asymptomatic children (as they could possibly be parasite carriers). Similarly, those who experienced at least one clinical malaria attack were classified in another group as uncomplicated malaria. Clinical and parasitological data for each child who had uncomplicated malaria were obtained from the longitudinal cohort study for malaria surveillance (Ndounga *et al*, unpublished). Those data included number of malaria attacks or episodes, parasite density and axillary temperature values.

Statistical analysis was performed using binary logistic regression model with Intercooled Stata v. 9.1 (STATA Corporation, Texas, USA) to determine the differences in allele frequencies and genotype distributions between uncomplicated malaria and asymptomatic children adjusted for age and gender. The level of significance was set at P<0.05. In addition, genetic models such as allelic, dominant and recessive were examined to evaluate the contributions of the genotype and allele frequencies using STATA. Kruskal-Wallis tests were employed to analyse the relationship between genotype distributions and the number of episodes as well as the risk of parasite density levels in the group of children with uncomplicated malaria. Genotype or haplotype frequencies were determined by simple gene counting and by using the expectation-maximum (EM) algorithm. The significance of deviation from Hardy-Weinberg equilibrium was tested using the random-permutation procedure as implemented in the Arlequin v. 3.5.1.2 software [22]. Linkage disequilibrium (LD) analysis was performed using Haploview V. 3.2 program.

## Results

### Characteristics of the study participants

Of the 323 children enrolled in the cohort study for malaria surveillance (Ndounga *et al*, personal communication), samples from 317 children were available for the present study. All the 317 children were successfully followed up during one year, and among them, 179 had at least one clinical malaria episode (forming the group of uncomplicated malaria children) whereas 138 did not experience any clinical malaria (forming the group of asymptomatic children). A total of 297 episodes were recorded without any case of recrudescence. No case of severe malaria was recorded and *P. falciparum* was the only *Plasmodium* species detected in children with uncomplicated malaria. Characteristics of recruited children are summarized in Table 2.

**Table 2:** Baseline characteristics of recruited children

**Characteristics**	**Mild malaria (n=179)**	**Asym (n=138)**	***P value***
Mean age (year)	4.73 ± 2.51	3.84 ± 2.56	NS
Sex ratio (Male/Female)	1.16 (96/83)	1.16 (74/64)	NS
Parasite carriers (%)	21 (11%)	10 (7.24%)	NS
Hb AA carries (%)	148 (82.69)	109 (79%)	NS
Hb SS carriers (%)	31 (17.31%)	29 (21%)	NS

Hb: haemoglobin; NS: non-significant; Unc malaria: uncomplicated malaria;

Asym: asymptomatic.

### Contribution of regulatory gene polymorphisms to uncomplicated clinical malaria

All the analysed SNPs did not deviate from Hardy-Weinberg equilibrium in each group. Genotype distributions and allele frequencies of these SNP variants are summarized in Table 3. Two novel SNPs were identified in the promoter region of *IL10RA* at the position -*163T/G* (ss491228441) and at the position -*163C/T* (ss491228440) in the promoter region of *TNFRSF18* gene, respectively. The identified novel SNPS were submitted to the SNPper database and appropriate SNP #ss IDs were received. The new SNP IDs for -*163T/G* of IL10RA and -*163C/T* variants are rs199832262 and rs150621809 respectively. Association analysis revealed that genotype *rs3024944CC* of *STAT6* gene was observed more in children who did not have clinical malaria episode during the follow up (asymptomatic group) compared to uncomplicated malaria group (Adjusted OR: 0.17, 95%CI: 0.05-0.55, P=0.003). The minor allele *rs3024944C* of *STAT6* gene was associated with decreased risk of clinical uncomplicated malaria (Adjusted OR: 0.57, 95%CI: 0.35-0.91, P=0.019). Interestingly, the heterozygous genotype *ss491228441TG* of the *IL10RA* was also found to be more frequent in asymptomatic children and the homozygous ss491228441TT genotypes were observed to be a risk factor (Adjusted OR: 6.35, 95%CI: 1.68-23.9, P=0.006). Also it was revealed that the minor allele *ss491228441G* of the *IL10RA* gene, despite it low overall frequency in Congolese children, was significantly more frequent in asymptomatic children compared to uncomplicated malaria group (Adjusted OR: 0.16, 95%CI: 0.04-0.61, P=0.007). The other regulatory polymorphisms in the *FOXP3, IL10RB* and *TNFRSF18* loci do not reveal any significant associations either at allele, genotype or at haplotype levels.

**Table 3:** Distribution of gene variants in children from the group of uncomplicated malaria and that of asymptomatic children

**Gene Variant**		**Unc malar (n=179)**	**Asym (n=138 )**	**OR (95%CI)**	***P *value**
***IL10RA ss491228441 T/G***	Genotypes				
	TT	176 (0.98)	125 (0.91)		
	TG	3 (0.02)	13 (0.09)		
	GG	0 (0)	0 (0)		
	Allele				
	T	355 (0.99)	263 (0.95)	Reference	
	G	3 (0.01)	13 (0.05)	**0.16 (0.04-0.61)**	**0.007**
	Dominant				
	TT	176 (0.98)	125 (0.91)	**6.35 (1.68-23.9)**	**0.006**
	TG+GG	3 (0.02)	13 (0.09)		
	Recessive				
	GG	0 (0)	0 (0)	NA	
	TT+TG	179 (1.0)	138 (1.0)		
***STAT6 rs3024944 G/C***	Genotypes				
	GG	145 (0.81)	106 (0.77)		
	GC	30 (0.17)	17 (0.12)		
	CC	4 (0.02)	15 (0.11)		
	Allele				
	G	320 (0.89)	229 (0.82)	Reference	
	C	38 (0.11)	47 (0.18)	**0.57 (0.35-0.91)**	**0.019**
	Dominant				
	GG	145 (0.81)	106 (0.77)	NS	
	GC+CC	34 (0.19)	32 (0.23)		
	Recessive				
	CC	4 (0.02)	15 (0.11)	**0.17 (0.05-0.55)**	**0.003**
	GG+GC	175 (0.98)	123 (0.89)		

NA: not applicable; NS: not significant; Unc malar: uncomplicated malaria; Asym: asymptomatic, Odds ratio and *P* value were calculated by using binary logistic regression model and adjusted for age and gender.

**Figure 1:**
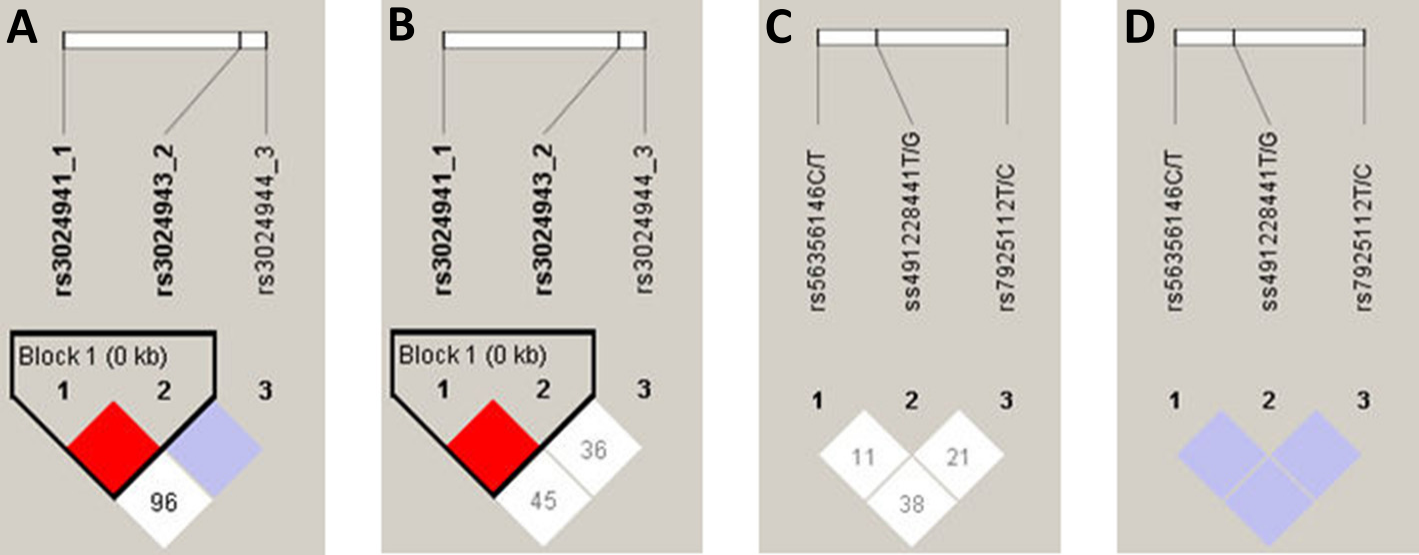
**Linkage disequilibrium of studied variants in asymptomatic and uncomplicated malaria.** Linkage disequilibrium pattern of studied SNPs in promoter regions of *STAT6* in uncomplicated malaria **(A)**; *STAT6* in asymptomatic children **(B)**; *IL10RA* in uncomplicated malaria **(C)**; and, *IL10RA* in asymptomatic children **(D)**. At the top, the SNPs are shown according to their succession from the promoter region of studied gene. Empty squares indicate a high degree of LD (LD coefficient D’ = 1) between pairs of markers, numbers indicate the D’ value, red squares indicate strong LD, purple and white squares indicate lower degree of LD with LOD <2. The haplotype block is outlined by a solid line.

The reconstructed haplotypes of *STAT6* (rs3024941/rs30240943/rs3024944) and *IL10RA* (rs56356146/rs7925112/ss491228441) were observed as a susceptibility factor between both studied groups and are presented in Table 4. In total, five different haplotypes were observed for *STAT6* locus, of which the major haplotype *CGG* was marginally significant with more prevalence among children who presented at least one clinical episode (uncomplicated malaria) (Adjusted OR: 1.4, 95%CI: 1.0-2.0, P=0.05). Similarly, five haplotypes were observed for the *IL10RA*, and the minor haplotype *CTG* was found to be associated with protection against the disease (Adjusted OR: 0. 16, 95%CI: 0.04-0.61, P=0.007). Different haplotypes of the other genes were not found to be significantly distributed between both groups of children. The linkage disequilibrium pattern of studied loci in promoter regions of *STAT6* and *IL10RA* are presented in Figure 1.

**Table 4:** **Distribution of *STAT6 *and *IL10RA *haplotypes**

**Gene ID**	**Haplotypes**	**Unc. Malaria n=358(%)**	**Asymptomatic n=276(%)**	**OR (95%CI)**	***P *value**
***STAT6****(rs3024941/ rs3024943 /rs3024944)*	CGG	276 (0.771)	193 (0.699)	**1.4 (1.0-2.0)**	**0.054**
	GAG	43 (0.121	36 (0.130)	NA	NS
	CGC	38 (0.106)	43 (0.156)	NA	NS
	CAG	1 (0.002)	0 (0)	NA	NS
	GAC	0 (0)	3 (0.011)	NA	NS
	CAC	0 (0)	1 (0.004)	NA	NS
***IL10RA****(rs56356146 /rs7925112 /ss491228441)*	CTT	284 (0.8)	209 (0.758)	NA	NS
	TTT	44 (0.12)	40 (0.145)	NA	NS
	CCT	26 (0.07)	14 (0.05)	NA	NS
	CTG	3 (0.008)	13 (0.047)	**0.16 (0.04-0.61)**	**0.007**
	TCT	1 (0.002)	0 (0)	NA	NS

NA: not applicable; NS: not significant; Unc malar: uncomplicated malaria; Asym: asymptomatic, Odds ratio and *P* value were calculated by using binary logistic regression model and adjusted for age and gender.

### Contribution of regulatory gene polymorphisms to clinical episodes and parasite density

**Figure 2:**
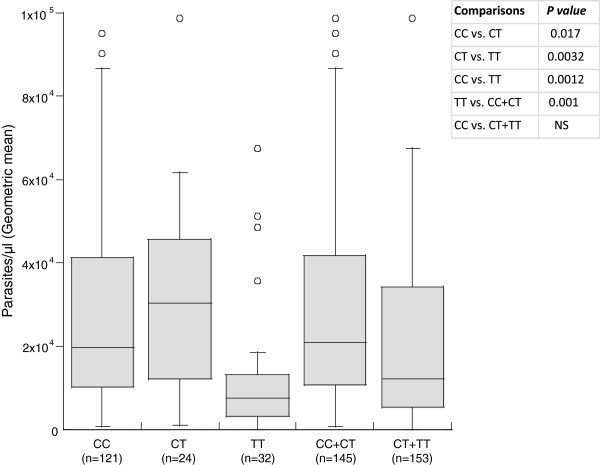
**Association of the *FOXP3 *rs11091253 SNP with parasite density levels.** Note. *P* values were calculated by Kruskal-Wallis test. Shown *P* values are corrected for multiple comparisons.

**Figure 3:**
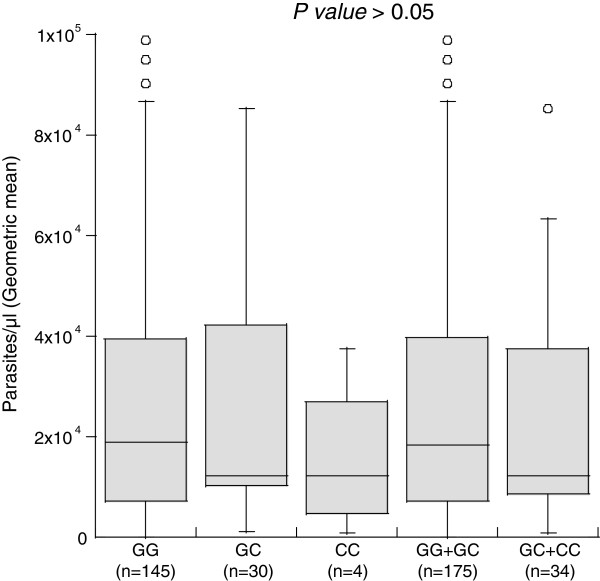
**Association of rs3024944 of *STAT6 *and parasite density levels.** Note. *P* values were calculated by Kruskal-Wallis test.

The effects of each single SNP variant on the number of clinical malaria episodes were investigated by simple comparison of genotype and allele distributions between individuals who experienced one malaria attack and those who experienced two or more malaria attacks. No association was found between the investigated SNPs and the number of malaria attacks. However analyses of the effect of individual single nucleotide polymorphisms on parasite density levels (Kruskal-wallis tests) showed an association between the *FOXP3* rs11091253 variant and *P. falciparum* parasitaemia. More precisely, patients carrying the rs11091253*CT* genotype harboured higher parasite densities compared to those carrying the rs11091253*CC* or rs11091253*TT* genotype (Figure 2). These results also showed that patients having the *STAT6* rs3024944*CC* genotype, which was correlated with protection against uncomplicated malaria, were found to have low levels of infection than those with *G/G* (not significant) (Figure 3).

## Discussion

Malaria has exerted selective pressure on the human genome that has led to changes, such as haemoglobin S which confers some resistance against severe malaria [23]. Understanding the mechanisms leading to increased susceptibility or resistance against the disease would assist in the elaboration of better tools to fight the disease. In this search, immune responses that are on the front of the defence are particularly investigated. Tregs are suggested to be central in the control of *P. falciparum* infection. Therefore, investigation of human polymorphisms in genes associated with Treg activity may underlie both susceptibility to infection and level of Treg expression.

The present study evaluated the contribution of promoter SNPs of *FOXP3*, *IL10RA*, *IL10RB*, *STAT6* and *TNFRSF18* genes in *P. falciparum* infection outcome among Congolese children. Earlier studies have identified some regulatory SNPs on the promoter loci of these five genes from 40 unrelated Gabonese individuals who had earlier infection episodes with various parasites, including *P. falciparum*[14,19-21]. These SNPs were then validated for their allelic gene expression using transient transfection assays. Some of the identified were already pre-described, while others were newly discovered. All the SNPs selected for the current investigation were found to alter expression of their respective gene [14,19-21]. Therefore any possible correlations of these SNP variants with resistance or susceptibility to mild malaria attacks and/or the control of *P. falciparum* infection levels were evaluated.

The results obtained in this study demonstrated that the *STAT6 rs3024944C/C* genotype was significantly more prevalent in asymptomatic children. This suggests the contribution of this genotype to protection of Congolese children against mild clinical malaria. The SNP rs3024944 is located in a transcription factor-binding site TCF-1 which is a member of family of genes with homology to high mobility group I (HMG) proteins [24]. TCF-1, in part with β-catenin, plays a pivotal role in T cell activation and is specifically expressed in T lymphocytes [25,26]. And the fact that in the uncomplicated malaria group, children with the minor allele *rs3024944C* have low parasite densities compared to those having the major allele *rs3024944G*, that speculates that the *rs3024944C/C* variant directs activation of T cells in response to *P. falciparum* infection, thus contributing to protection against the disease through parasite elimination. In haplotype level, the major haplotype CGG was found to be correlated with the marginal risk of uncomplicated malaria. This emphasizes the role of the SNP rs3024944 on susceptibly/resistance to uncomplicated malaria among Congolese children. This is the first study that shows the correlation between a promoter SNP *STAT6* rs3024944 and protection against uncomplicated malaria. However, in previous investigations, the promoter SNP rs324013 was found to be associated with the control of *S. haematobium* infection levels in a Dogon population in Mali [17]. Similarly, the variant rs324015 was associated to the control of *A. lumbricoides* infection levels in Chinese children [18].

Two novel promoter SNPs were identified including, ss491228441 and ss491228440 of *IL10RA* and *TNFRSF18* respectively. In both cases the frequency of the minor allele was low. Meanwhile the minor allele *ss491228441G* of *IL10RA* appeared to contribute to protection against uncomplicated malaria. Interestingly, the haplotype *CTG* involving this minor allele *ss491228441G* was significantly more present in asymptomatic children. This provides an additional evidence of the involvement of this allele in protection against uncomplicated clinical malaria. However, larger association studies in different malaria endemic areas including children and adults as well as functional studies of this ss491228441 SNP variant would be of great interest to verify the current observations.

The data also showed the association between the carriage of the *FOXP3* rs11091253 SNP variant and *P. falciparum* parasite density levels. In particular, the *rs11091253CT* genotype was found to be associated with high infection levels, whereas the homozygous for the minor allele, the *rs11091253TT* genotype, was associated with lower infection levels. This suggests that *FOXP3* contributes to the modulation of the immune response against *P. falciparum* infection. The SNP rs11091253 is located in a putative binding site for the transcription factors nuclear factor kappa B (NF-κB) and c-Rel, which is also a member of NF-κB family. These transcription factors have been demonstrated to be critical for regulation and transcription of *FOXP3*[27,28]. Therefore the differential binding properties of the promoter carrying the *rs11091253CT* genotype and that of the promoter carrying the *rs11091253TT* genotype may explain the associations observed in this study. One possible explanation may be that the binding of NF-κB and/or c-Rel with the promoter carrying the *rs11091253C/T* genotype results in upregulation of *FOXP3* which in turn leads to increased number of fully functional Tregs. Consequently, parasite multiplication is facilitated by suppression of either *P. falciparum*-responsive effector cells or pro-inflammatory cytokine response. This is in line with what was reported by previous study showing that upregulation of *FOXP3*, and CD4^+^CD25^+^ regulatory cells correlates with more rapid parasite growth in human malaria infection [7].

## Conclusion

To summarize, selected regulatory SNPs in the promoter region of *FOXP3*, *IL10RA*, *IL10RB*, *STAT6* and *TNFRSF18* genes have been investigated for possible association with uncomplicated malaria and high *P. falciparum* parasite density among Congolese children. These findings indicate that the *STAT6* promoter polymorphism rs3024944 is highly associated with protection against uncomplicated malaria, whereas the *FOXP3* promoter variant rs11091253 is more likely to be involved in the control of *P. falciparum* parasite density levels. In addition, a new SNP detected in the promoter region of *IL10RA* (ss491228441) contributes to protection against clinical malaria. These findings deserve to be further investigated in a larger population of children in the Republic of Congo but also in other malaria endemic areas.

## Competing interests

The authors declare that they have no competing interest.

## Authors’ contributions

MN designed and performed the field study. VTP designed and supervised the experiments. FKK performed the experiments. FKK, HVT, and VTP analysed data. VTP, FN, and AAA contributed materials/analysis tools. FKK, FN, and VTP wrote the paper. All authors read and approved the final manuscript.
